# Coping strategies in active and inactive men with prostate cancer: a qualitative study

**DOI:** 10.1007/s11764-021-01037-3

**Published:** 2021-04-09

**Authors:** David Michael Langelier, Colleen Jackson, William Bridel, Christopher Grant, S. Nicole Culos-Reed

**Affiliations:** 1 Faculty of Kinesiology, University of Calgary, Calgary, Canada; 2 Division of Physical Medicine and Rehabilitation, University of Calgary, Calgary, Canada; 3 Faculty of Medicine, Princess Margaret Cancer Centre, 550 University Avenue, Toronto, Ontario M5G 2A2 Canada; 4 Department of Psychosocial Resources, Tom Baker Cancer Centre, Calgary, Canada; 5 Department of Oncology, Cumming School of Medicine, University of Calgary, Calgary, Canada

**Keywords:** Body image, Exercise, Masculinity, Movement, Prostate neoplasms, Qualitative

## Abstract

**Purpose:**

Prostate cancer can result in a shift in the way men perceive their masculinity. Despite the interest in exercise as a treatment strategy to address masculinity concerns, there is insufficient information about how perceptions may differ in active and inactive men. The aim of this study was to explore how exercise might influence self-perceptions of masculinity in men across the exercise continuum (from active to inactive) and in men receiving different forms of treatment for their prostate cancer, including androgen deprivation therapy.

**Methods:**

Individual, semi-structured interviews were conducted with 15 men. Ten men met aerobic and/or resistance guidelines and were considered active, while five men, considered inactive, reached neither guideline. This study used a grounded theory approach to data analysis, examining masculinity issues in active men and compared them to inactive men.

**Results:**

Redefining masculinity emerged as an overarching theme. Subthemes were the various coping strategies men used to redefining masculinity and directly related to their exercise habits. Coping subthemes included re-establishing control, tapping into competition, remaining socially connected, rationalization, and acceptance.

**Conclusions:**

In the active men, dominant coping strategies achieved from exercise included control through active participation, acceptance, competition, and leadership. In inactive men, control was observed with knowledge-seeking behaviors, rationalization, and acceptance.

**Implications for Cancer Survivors:**

A tailored approach to exercise counseling based upon specific masculine traits and motivations could lead to improved exercise engagement.

**Supplementary Information:**

The online version contains supplementary material available at 10.1007/s11764-021-01037-3.

## Introduction

Concerns about one’s masculinity following prostate cancer diagnosis and treatment are prevalent and have been associated with numerous physical, emotional, and social changes [1–3]. Perceived changes to masculine identity have been expressed by some survivors to be the most challenging side effect experienced [4]. Studies have shown men desire a return to pre-morbid masculinity levels when traditional traits of masculinity, such as control and erectile function, have been lost [5, 6].

Preliminary evidence suggests exercise can play a pivotal role in the treatment of masculine identity by addressing common side effects (e.g., body composition, sexual function) that contribute to low masculinity levels [7–10]. A recent systematic review of the qualitative literature found masculinity was influenced by increased levels of camaraderie secondary to shared sense of impairment or a common goal of “battling cancer”; providing an environment focusing on valued male traits; providing distraction from one’s mortality or impairment; increasing participant’s sense of control; improving body composition, thereby re-establishing similarity between idealized masculine phenotype and what they see in the mirror; and re-establishing a belief in their ability to succeed or develop a new identity [11]. However, most of this research to date has focused only on men undergoing androgen deprivation therapy (ADT) [7, 12–14] and primarily on men currently exercising. The aim of this study was to explore how exercise might influence self-perception of masculinity in men across the exercise continuum (from active to inactive) and in men receiving different forms of treatment for their prostate cancer, including androgen deprivation therapy.

The present exploratory study interviewed men with prostate cancer to understand the intersection between masculinity and levels of exercise. We specifically recruited two distinct participant groups based on exercise levels, those meeting either aerobic or resistance exercise guidelines (i.e., active) and those failing to meet either guideline (i.e., inactive), to ascertain whether perceptions of masculinity differed and why.

## Methods

### Participants

Participants were recruited from the Tom Baker Cancer Centre and local cancer exercise programs. Eligible participants had histologically confirmed adenocarcinoma of the prostate, had minimum 6-month duration of disease, were fluent in English, and indicated on preliminary questionnaires they were willing to participate in an interview. No restrictions were placed on treatment history including receiving or previously exposed to androgen deprivation therapy. Patients provided written consent prior to enrolment and were reimbursed for parking. Ethical approval of the study was granted by Health Research Ethics Board of Alberta—Cancer Committee (HREBA.CC-16-0625).

### Procedure

This study was part of a cross-sectional study that examined exercise levels and patient-reported levels of masculinity, body image, and quality of life [15]. Using the modified Godin Leisure Time Exercise Questionnaire [16], men able to achieve guideline levels of either aerobic or resistance activity (i.e., >150 min of moderate-to-vigorous aerobic exercise or > 2 days/week of resistance exercise, respectively) were considered active. Men exercising at levels below both aerobic and resistance thresholds were considered inactive [17]. These definitions follow the American College of Sports Medicine (ACSM) guidelines for cancer survivors [17]. Men who have previously participated in an exercise programming but did not continue with levels sufficient to be classified as active at the time of the study were considered inactive.

**Fig. 1:**
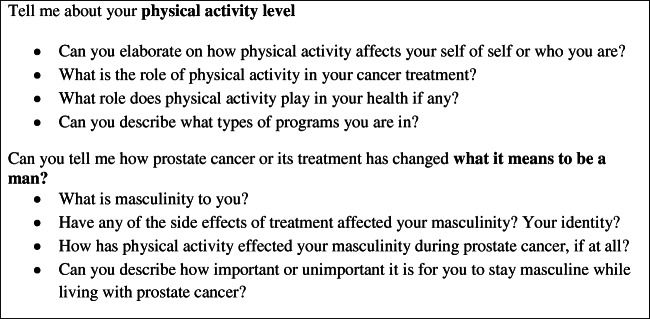
Semi-structured interview guide

The first author, a medical doctor and trained researcher with experience interviewing oncology patients, conducted all interviews in person or by phone. The semi-structured interview included open-ended questions designed to encourage discussion between the participant and the interviewer (Fig. 1). No participant-researcher relationship existed prior to the interviews. Questions were informed by prior research and the clinical expertise of the first and last authors. The study was conducted in accordance with the Consolidated Criteria for Reporting Qualitative Research [18].

### Analysis

Interviews were audiotaped, de-identified, and transcribed verbatim. Each interview was approximately 60 min in length; as themes emerged, probing questions were asked to ensure men were attributing specific coping strategies to their exercise habits and perceptions of masculinity. The first author verified the accuracy of all transcripts, and additional field notes were made to capture key points, track biases, and provide cues for follow-up. Data were individually coded by the first and second authors line-by-line in an analysis software (NVivo) [19] using thematic content analysis [20]. Both coders utilized a constant comparisons method to first familiarize themselves with the material and then identify unique concepts, hierarchical categories, and overarching themes that emerged from the data. Coders met regularly to check themes against one another and agree upon specific terminology, criteria, and placement within the hierarchy. This process was iterative and occurred several times until agreement was achieved. In the case where the first and second authors were unable to come to a consensus, the last author assisted in making a decision. Overall, all researchers felt there was consistency among the themes from active and inactive groups and agreed upon the key differences observed.

## Results

**Fig. 2:**
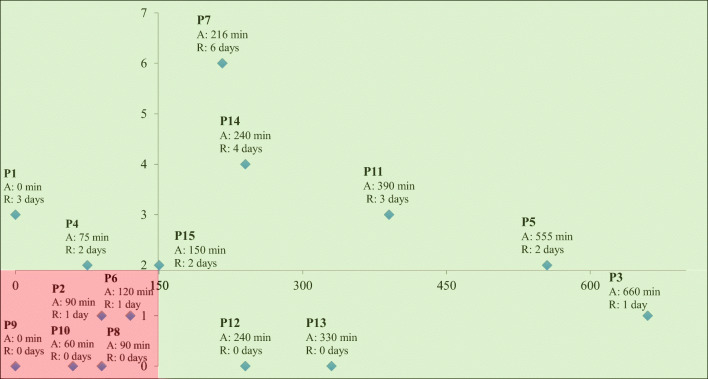
Distribution of participants based on participation in aerobic (*x*-axis) and resistance (*y*-axis) activity. P, participant; A, minutes of aerobic activity; R, days per week of resistance activity; green/light shaded region, men classified as active based upon meeting guidelines levels of aerobic and/or resistance activity; red/dark shaded region, men classified as inactive based upon failing to meet both aerobic and resistance guideline levels of activity

Table 1 depicts the demographic details of active and inactive participants. Of the 15 participants interviewed, *n* = 10 met the criteria to be considered active, and *n* = 5 were classified as inactive. The mean age of participants was 64.9 years; all were Caucasian (100%); and the majority were married (86.4%), heterosexual (93.3%), and university educated (86.7%). Participant interviews revealed “redefining masculinity” as the single overarching theme of the study. Subthemes represented the various coping strategies active and inactive men used to redefine their self-perceived masculinity. Coping strategy subthemes included the ability for men to (1) re-establish control, (2) tap into competition, (3) remain socially connected, (4) rationalize, and (5) accept their new reality. The different subthemes represented the various ways men used or understood the role of exercise to cope with self-perceived changes regarding masculinity; they differed based upon whether men were active or not. Additionally, most subthemes were found to have multiple distinct strategies which could be distinguished based on participants’ exercise level. This hierarchy of classification was determined by multiple rounds of participant feedback, most specifically to ensure that no strategy should be included as a subtheme. Table 2 depicts the subthemes and strategies and illustrates the number of participants and the number of times items were mentioned. Figure 2 depicts the various volumes of aerobic and resistance activity of interviewed men.

**Table 1:** Participant characteristics

Characteristic	Active (*n* = 10)	Inactive (*n* = 5)
Age in years (mean, SD)	63.5 + 9.3	67.8 + 10.4
Education (*n*, %)		
High school	2 (20)	0 (0)
University	4 (40)	3 (60)
Graduate	4 (40)	2 (40)
Marital status (*n*, %)		
Married	9 (90)	4 (80)
Not married	1 (10)	1 (20)
Ethnicity (*n*, %)		
Caucasian	10 (100)	5 (100)
Employment (*n*, %)		
Full time	2 (20)	0 (0)
Part-time	3 (30)	0 (0)
Retired	4 (40)	5 (100)
Disability	1 (10)	0 (0)
Sexual orientation (*n*, %)		
Heterosexual	9 (90)	5 (100)
Homosexual	1 (10)	0 (0)
Months since diagnosis (mean, SD)	42.0 + 47.0	93.2 + 77.5
Cancer stage (*n*, %)		
1	1 (10)	0 (0)
2	4 (40)	3 (60)
3	4 (40)	1 (20)
4	1 (10)	1 (20)
Treatment (*n*, %)		
Prostatectomy	10 (100)	5 (100)
Radiation	3 (30)	2 (40)
Chemotherapy	2 (20)	2 (40)
Androgen deprivation therapy	4 (40)	4 (80)

**Table 2:** Theme and subtheme expression by active (meeting aerobic and/or resistance exercise guidelines) and inactive (failing to meet either aerobic and resistance guidelines) men with prostate cancer by number of participants and number of times mentioned (*n* = 15)

Theme: redefining masculinity				
	Active men (*n* = 10)		Inactive men (*n* = 5)	
Subtheme: coping strategies Subtheme strategies	Number of participants	Number of times mentioned	Number of participants	Number of times mentioned
Re-establish control				
Active participation and knowledge seeking	10	67	5	49
Learned resilience	7	19	1	2
Structure	6	13	0	0
Tapping into competition				
Internally focused	10	19	0	0
Externally focused	6	21	0	0
Remain socially connected				
Becoming a leader/teacher/role model	9	29	2	8
Embracing femininity	6	10	3	7
Upward and downward social comparisons	10	13	5	16
Rationalization				
Attribute impairment to aging	6	16	5	26
Attribute dysfunction to diagnosis or treatment	10	16	5	15
Blame on partner	5	6	3	7
Acceptance	10	27	5	18

### Subtheme 1: re-establish control

For active men, re-establishing control occurred by actively participating in their own care; by appreciating their resilience and ability to recover from side effects; and through “structured or regimented” activities, like exercise. These strategies differed from inactive men who primarily gained control through knowledge-seeking behaviors. Inactive and active men also differed in their motivations for exercise, demonstrating different understandings of the potential ways exercise could improve wellness.

#### Active participation and knowledge-seeking behavior

Active men exercised as a way to participate in the “battle against cancer” or resist the side effects imposed upon them by treatment.

I feel like I’m beating it by doing what I’m doing. As long as I keep doing [exercise], I’m going to keep beating it. I think that has a big impact on how you feel, and I think, beating the cancer. (P_15_, Active)

Active men had clearly established a connection between exercise and control. They were motivated to exercise because it was one of the few behaviors completely under their control. Additionally, active men were motivated to exercise to restore or maintain physical functioning. This contributed to improved masculinity through empowerment and facilitated independence from family/friends by allowing them to participate in household tasks they previously relinquished.

Off the top of my head, probably exercise mainly for taking back or holding onto what the cancer took from me. You have to work harder to hold onto what remains. That makes me feel empowered as a man. (P_3_, Active)

It was important for men who were active to not merely engage passively with treatments (i.e., taking medications or choosing between various treatment options) but to do something physical as part of their active participation.

Keeping a bit more fit makes you feel good and makes you feel like you, that you are in control more than just relying on the pill from the doctors (P_7_, Active)

In contrast, for the inactive men, “active” participation was observed to include decision-making and knowledge-seeking behaviors. Men described an internal pressure to become knowledgeable about their illness through activities such as attending support groups; through reading books and magazines or surfing the Internet; and by tracking their prostate specific antigen (PSA). Treatment decisions included choosing a form of treatment (e.g., radical prostatectomy versus robotic) or deciding to escalate treatment because of the inherent feeling that they must “do something” (e.g., pushing their oncologist for surgery in low-risk disease).

I said to [the urologist], number one, get the cancer, that’s my priority, number two let’s hope the incontinence is not too bad…you can’t be prepared for the downsides but certainly treatment decisions you can take the bull by the horns. (P_8_, Inactive)

Some inactive men understood the importance of exercise, but it appeared that the full range of benefits was often not fully realized. For example, inactive men acknowledged they should be participating in higher levels of exercise for increased longevity and improvements in healing and comorbidity as primary reasons. Inactive men were also more likely to receive pressure to exercise from external sources (e.g., physicians and family) or used exercise as a distraction from side effects.

Exercise is a great way to just get out of the house and walk away from things. If I’m going to cry, which is an uncomfortable and uncharacteristic thing to do…either sitting in another room or going out is the best. (P_10_, Inactive)

#### Learned resilience

Resilience embodied the capacity for men to remain connected to their pre-cancer level of function. This was observed more frequently in active men and was strongly associated with a maintenance of employment or position. This was perceived by active men as one of the main mental health benefits associated with exercise and a way control was regained from the “chaos of cancer” or its treatment.

For me, mentally that was my fight. I wasn’t going to let [cancer] take over my life, so I just went to work…This way I was still maintaining some control over my life as I saw it then. If you’re getting someone on a fitness program before, it’s going to help them deal with stress, and to think more logically. (P_13_, Active)

While exercise capacity could also be threatened by changes to the body from treatment, exercise provided the chance for recovery. By observing improvements in their health following exercise, positive coping and resiliency were reinforced for those exercising.

#### Structure

Exercise represented a structured activity. It occurs over a particular period of time, involves specific movements, and requires certain technique. Expressions of structure contributed to an increased sense of control and appeared exclusively among aerobically active men who valued a regimented program.

So when I exercise I put, P, for plan, the resistance, the number of repetitions, the number of sets and then, A, the actual what I did…it’s only an hour, but it’s three hours of exercise a week, plus an hour or yoga, so I’m four hours a week of scheduled exercise. (P_13_, Active)

### Subtheme 2: tapping into competition

The ability for survivors to compete within themselves or against other survivors was a major coping strategy for active men only. Competition occurred either intrinsically from “an internal fire” or externally from setting new goals through observation of peers. Instead of tapping into competition, inactive men tended to utilize coping strategies of rationalization and social connectedness.

#### Internally focused competition

Active men were observed to have an overwhelming internal drive to improve their general well-being with exercise. They often intentionally set personal exercise goals beyond those measured by qualified exercise professionals, igniting competition within. These personal goals were based on the participant’s barometer of his capabilities.

When I’ve done a good workout I feel like I’ve lifted what I can lift, I’ve pushed what I can push, I’ve gone the extent that I want to go with cardio, I just feel good. (P_5_, Active)

Men participating in exercise classes also celebrated improvements in objective outcomes collected by the researchers (i.e., fatigue scores, degree of flexibility, strength, and overall fitness) as a measure of success. This internal achievement did not require the presence of others.

I gained, even in the first 12 weeks… six centimetres on my reach. Small measurable gains [are] important! One last run, lasting an hour longer. (P_13_, Active)

#### Externally focused competition

This was observed when men began comparing themselves to others to set personal goals or feel a sense of achievement. Active men were frequently reporting a struggle post-treatment to establish new and realistic exercise goals. By observing active peers, men could increase their own exercise levels by visualizing competition alongside or against them.

I’m always competitive, yes, even though I shouldn’t be, but if there’s someone in my age group with prostate cancer, if I’m going to run against them, I will try and keep up to them. (P_15_, Active)

By exercising in a social setting, active men were able to tap into a competitive spirit. The exercise environment appeared to facilitate competitive nature and subsequently increase further exercise participation, improve sense of coping, and increase feelings of masculinity.

### Subtheme 3: remaining socially connected

In order to cope with prostate cancer, active and inactive men began to establish new social networks or attempted to redefine the previous ones. Relationships included those with family, friends, or peers. In general, men in the active group expressed more statements supportive of building social relationships than men who were inactive. Social connections commonly took the form of leadership positions; upward and downward social comparisons in order to provide a frame of reference for their impairments; and using what are typically construed as feminine traits or attributes as a way into different social networks.

#### Becoming a leader/teacher/role model

The most common reaction after developing prostate cancer was for men to experience a shift in their identity, role, or perception of their masculinity. Men on either side of the exercise spectrum were observed re-evaluating their involvement in activities and gravitated toward roles emphasizing leadership and teaching to improve feelings of masculinity. For active men, leadership activities were often easily found within exercise as it offered opportunities for this process to occur naturally, whereas inactive men often took leadership roles within administrative or educational settings.

I’ve coached people, I’ve run running groups and stuff and that’s why I was participating in [exercise]. I can help people if they have a problem with [exercise] and prostate cancer. If you are a good example, then people learn from that. (P15, Active)

So we advocate for men and prostate cancer and, of course, we have monthly meetings every second Tuesday so I started to go to those, and I knew one of the participants there and so I ended up getting on the board of directors. (P8, Inactive)

Inactive men who had never previously participated in a formal exercise program also expressed achievement by mentoring newly diagnosed males. The capacity to offer wisdom and provide encouragement helped expand their social network, create a feeling of purpose, and improve coping.

#### Upward and downward social comparisons

The ability to compare oneself to others allowed men to appreciate their own health. This subtheme differed from external competition (i.e., tapping into competitiveness) because it did not result in a man trying to obtain a tangible goal or outcome but was used instead to cope with his own impairment (e.g., limited exercise tolerance or incontinence). Men also looked beyond other men with prostate cancer to friends and family with health conditions or younger generations with medical problems to feel better about their impairments. Self-evaluating their physical, emotional, or functional impairments as being less severe often led to enhanced feelings of masculinity, body image, and coping.

I work down at the Y and some of the guys are older than me or as old as I am, and you see them and they got breasts too. They don’t have the cancer. I’ve got the cancer, so seeing those changes to [my body] doesn’t bother me much. (P_15_, Active).

#### Embracing femininity

For both active and inactive men, a softening of the body, hot flashes, and breast development were unwelcome occurrences. There did not appear to be a major difference in how active or inactive men embraced this new femininity with respect to exercise. However, for men who were accustomed to traditionally “masculine” resistance training, participation in activities like yoga represented openness and an opportunity to explore new activities including ones defined as “feminine.”

I’ve become more open to these sorts of things…going into the showers after class, there’s still a lot of guys there that have just been up in the gym pumping iron and doing whatever we do up there, and we come in with our yoga mats, and you can see some of them are inquisitive, like, what are these guys doing?.... and I’ll say to them you guys really need to go to a yoga class to understand what it does, the way it improves your flexibility, stamina, [and] core strength. It’s completely different than what I’m used to but effective. (P_4_, Active)

In other men, changes perceived as feminine were embraced or justified by viewing emotionality as a strength. Consciously many participants appeared to embrace body image and perceived changes to their masculinity, and exercise provided the outlet for unmodifiable changes to be embraced.

### Subtheme 4: rationalization

Rationalization involved men making excuses or attempting to “rationalize” their feelings and/or impairments to convince themselves that their problems were tolerable or the fault of someone else. Placing blame on the natural aging process, dissociating side effects from identity, and attributing relationship changes to the faults of their partner were the strategies observed. These helped men to maintain elements of their masculinity and emerged more strongly from the inactive men.

#### Attributing impairment to aging

All five inactive men had convinced themselves that perceived masculinity changes experienced from prostate cancer would have inevitably occurred over time. For the active men, however, age served to rationalize how further performance gains were not possible with additional exercise.

I do feel like less of a man but my body’s not working the way it used to, and I equate that to my medical history and to my age. (P_1_, Inactive)

You look at somebody and you ask them at my age, can you do any push-ups? No! So even if I can’t, can’t get more through exercise, its okay I guess. (P_12_, Active)

#### Attributing dysfunction to diagnosis or treatment

When experiencing side effects of prostate cancer, both active and inactive men were able to disassociate masculine identity from treatment complications. This coping strategy was observed to protect masculinity even when men were experiencing uncharacteristic side effects (e.g., feeling ill, having scars, becoming incontinent). Men being instructed on what side effects to expect with treatment was an important prerequisite for mentally separating self from side effect.

I was warned I would be leaking for a while, this allowed me to separate it and have no, no bearing on my masculinity whatsoever. (P_1_, Inactive).

#### Blame on partner

Both heterosexual and homosexual men, regardless of activity levels, frequently commented on their partner’s ill health, age, and/or menopause as the primary reasons their sexual relationship had changed. This occurred even after acknowledging the negative changes to their own sexuality, masculinity, and body image.

My wife had already started early menopause, so we were just sort of, so [my erection] wasn’t a big threat to our sex life. (P_2_, Inactive)

By men inferring their partner was disinterested in sex, a major part of their sexual anxiety and the threat to their masculinity could be reduced. Additionally, men appeared to believe that they were supportive by honoring their partner’s “wishes” for companionship versus penetrative intercourse. Therefore, the blame placed on the partner for the male’s impairments helped rationalize their situation and facilitated coping while improving their self-perceived masculinity.

### Subtheme 5: acceptance

Acceptance represented an individual’s assent to the reality of living with cancer. Every individual regardless of activity levels made explicit statements about acceptance and it appeared critical in order to stabilize their feeling of masculinity loss and face their fear of their own mortality.

I do feel like less of a man but have learned to be comfortable in my own skin. I’ve become less embarrassed about having had cancer, it may come back…I’m going to have to move on after that. Exercise can, it can help. (P_1_, Active)

Acceptance involved men recognizing some impairments were permanent. Men often did not attempt to resist these impairments from treatment because the “alternative (i.e., death) could be worse.” Resisting treatment to preserve masculinity was observed as too high a cost for both active and inactive men. When men accepted their situation, they often required a shift in their priorities to accept new personality traits as more valuable. For both active and inactive men, they prioritized items like family and friends. This shift was observed as positive, with many expressing thanks for the closer bonds with loved ones and change in perspective.

All things considered, I am a richer person for [getting prostate cancer]. I’m not complaining that I have it. In terms of a new sense of priority in terms of who is important to me. (P_7_, Active)

## Discussion

Masculinity has been described as the interaction of numerous biopsychosocial factors [21]. Men at highest risk of developing prostate cancer (i.e., those over the age of 65) largely grew up learning and performing contextually specific hegemonic forms of masculinity [22], which dictated how “masculine” men should look, think, feel, and act [23, 24]. In its idealized form, hegemonic masculinity in the Canadian context has been inflexible and strictly emphasized traits of independence, emotional withdrawal, competitiveness, and control, while also instructing men to portray a body image consistent with sexual potency, muscularity, and functionality to be a provider [21, 25, 26].

While hegemonic forms of masculinity may predominate in Western societies and in the generation of men at highest risk of prostate cancer, other non-hegemonic or flexible interpretations of masculinity exist [27–30]. However, even flexible interpretations of masculinity can share hegemonic values or roles that can be directly impacted by prostate cancer or its treatment [23, 24, 31–33]. This is perhaps most exemplified in men treated with ADT, whereby the anti-testosterone effect of ADT can result in significant reductions in lean muscle mass, predispose men to central adiposity, and heighten fatigue symptoms [34]. Treatment-associated threats to hegemonic traits have been found to significantly reduce self-perceived masculinity and are associated with reductions in quality of life [30, 35]. Therefore, interventions such as exercise which may directly and indirectly address masculinity concerns, beyond the known improvements to fitness, health, and wellness, have become increasingly important.

The need to establish control over the cancer or its impairments emerged as a dominant coping strategy as men attempted to redefine self-perceived masculinity impacted by prostate cancer. In a recent review by Lashbrook et al., men with prostate cancer tended to implement problem-focused (i.e., planning, knowledge seeking, and situational withdrawal) versus emotion-focused (i.e., social support) coping strategies [36]. While our study is generally supportive of these assertions, our findings suggest exercise levels may correspond to particular problem-focused approaches in men with prostate cancer. Active men saw exercise as a self-management tool and manifestation of their fighting spirit to gain control over their illness, whereas inactive men tended to use problem-focused control strategies of knowledge seeking, withdrawal, and social support outside the exercise environment. Previous studies examining exercise behaviors in men treated with ADT have observed similar correlations between exercise and control but have also found spousal support could positively influence exercise adoption which was not a key finding from our results [37].

Similar to control, the problem-focused approach of rationalizing impairments and flexibly interpreting one’s masculinity was generally supportive of previous studies demonstrating improvements in self-perceived masculinity and quality of life while exercising [5, 30, 35, 38, 39]. Furthermore, strategies of acceptance and optimism demonstrated by both active and inactive men in this study are well-known coping strategies in men with prostate cancer [6, 40]. For instance, Pascoe et al., suggested that acceptance could provide the strongest individual benefit for men with prostate cancer compared to other coping strategies [41].

To the authors’ knowledge, no other study has directly examined masculinity issues in men with prostate cancer with the consideration of exercise levels. This study suggests active men could be motivated to participate or stay involved with exercise by appealing to active participation, competition, or facilitating leadership opportunities. This differed from the experience of inactive men where rationalization, knowledge-seeking, and comparison strategies were predominately used to increase feelings of perceived masculinity. Additionally, for inactive men, the usefulness of exercise was more limited but could be motivated by physicians or family members and by appealing to its effect on longevity or comorbid health conditions.

### Limitations, strengths, and future directions

The sample of men in our study was relatively homogenous, and there was a relatively small number of men comprising the inactive versus active group. Additionally, the literature remains unclear about the relative importance of either form of exercise (i.e., aerobic or resistance) on self-perceptions of masculinity [15]. Qualitative research findings from this exploratory study could inform larger-scale quantitative or mixed methods studies that include a larger more diverse sample of men across various ethnicities, sexual orientations, and ages. Additionally, capturing men meeting resistance-only, aerobic-only, or combined aerobic and resistance exercise guidelines both on and off ADT would be necessary to explore further whether one exercise modality may be more influential to self-perceived masculinity and how this corresponds to ADT exposure. For instance, in a study by Keogh et al., distinct differences were observed in the motivations for exercise in men who had received ADT versus those who had not [42]. While men treated with ADT reported stronger engagement with resistance training to directly counteract the effects of muscle loss, reduced strength, and sense of masculinity, those without ADT exposure tended to find motivation for different reasons such as implementing exercise to help manage chronic medical conditions [42]. Future studies examining the role of ADT and specific exercise modalities (i.e., aerobic, resistance, or combined) could highlight important behavioral, treatment, and situational factors as they relate to an individual’s self-perceived masculinity to potentially improve exercise-related counseling. Finally, future qualitative studies examining masculinity issues prospectively in a pre-/post-trial format could offer insight into how coping may change or remain stable in men participating in various amounts of exercise. Future prospective trials incorporating validated measures of illness coping may also assist researchers in determining whether exercise can influence coping or whether it is the underlying coping strategy influencing exercise participation.

Despite some of these limitations, our study has several strengths. No previous work has included men outside of those on androgen deprivation therapy when examining the relationship between exercise levels and self-perceived masculinity. Additionally, by understanding the differences between how active and inactive men view exercise as it directly relates to masculinity, these results have the potential to provide impactful programming recommendations on how exercise could be presented or delivered differently to active and inactive men to help improve motivation.

### Implications for practice

The ultimate objective is to encourage men with prostate cancer to be more active, and this study has provided several meaningful implications for practice. For men who are active or want to be active, clinicians can reinforce exercise by recommending community programs that may foster specific masculine traits. For example, encouraging participation in prostate cancer sporting groups (i.e., recreational football), engaging in exercise trials that are purposefully measuring anthropomorphic details or fitness changes (i.e., skin fold thickness, degree of flexibility, one repetition maximum), or tailoring exercise prescriptions based on treatment exposure (i.e., strength centric program for men treated with ADT [42]) could further increase exercise participation. Alternatively, clinicians could assess and then advise a patient based on a particular coping strategy. Empowering the clinician with this information enhances the ability to provide targeted motivational counseling that will be more likely to foster successful behavior change.

A similar approach to increasing exercise participation could be used in inactive men. First, the importance of physician-to-patient education and providing a clear exercise prescription appear more valuable to inactive men compared to active men for increasing motivation to exercise. Numerous studies have supported these conversations as a way for the clinician to better understand the potential barriers (e.g., incontinence, avoidance of social exercise), which could then be effectively addressed with targeted therapy (such as pelvic physiotherapy or referral to urology) or referral to exercise programs that can address such barriers (such as one-on-one exercise programs) [42, 43]. Additionally, explicit exercise prescription may be able to shift the way inactive men “participate actively” to structured exercise versus engaging only in treatment decisions. Second, by encouraging men to engage in knowledge-seeking strategies, providing an environment where comparisons to other men can occur, and educating patients on the role of exercise on longevity, increasing general health and improving comorbidities may draw upon inactive males’ coping strategies. Understanding the coping strategies commonly employed by inactive men may allow for motivational counseling to appear in this group as well. This could include “Based upon other men who are less active like yourself, some men may exercise more if they knew exercise had the potential to improve your survival or help with your knee osteoarthritis.” Or “Based upon other men who are less active like yourself, participating in exercise programs can allow for you to see other inactive men similar to yourself starting to move more. This can help contextualize some of the challenges you are experiencing.” Overall, given the observed differences between active and inactive men, a tailored approach to exercise counseling that may draw on certain masculine traits and related motivations should be considered.

## Supplementary Information

ESM 1 (PDF 504 kb).

## Data Availability

Available upon request
